# Australia’s First Peoples: hunters of extinct megafauna or Australia’s first fossil collectors

**DOI:** 10.1098/rsos.250078

**Published:** 2025-10-22

**Authors:** Michael Archer, Kim Akerman, Larisa DeSantis, Blake Vermeer Dickson, Suzanne Hand, Lindsay Hatcher, John C. Hellstrom, Geraldine Jacobsen, Julien Louys, Gilbert James Price, Helen Ryan, Kale Sniderman, Kenny Travouillon, Jon Woodhead

**Affiliations:** ^1^ School of Biological, Earth and Environmental Sciences, University of New South Wales, Sydney, New South Wales, Australia; ^2^ University of New South Wales, Moonah, Tasmania, Australia; ^3^ Department of Biological Sciences, Vanderbilt University, Nashville, TN, USA; ^4^ Department of Anatomy, University of New South Wales, Sydney, New South Wales, Australia; ^5^ University of New South Wales, Margaret River, Western Australia, Australia; ^6^ School of Geography, Earth and Atmospheric Sciences, University of Melbourne, Parkville, Victoria, Australia; ^7^ Centre for Accelerator Science, ANSTO, Kirrawee, New South Wales, Australia; ^8^ Australian Research Centre for Human Evolution, Griffith University, Brisbane, Queensland, Australia; ^9^ School of the Environment, University of Queensland, Brisbane, Queensland, Australia; ^10^ Collection and Research Centre, Western Australia Museum Collections and Research Facility, Welshpool, Western Australia, Australia; ^11^ School of Molecular and Life Sciences, Curtin University, Perth, Western Australia, Australia

**Keywords:** extinction, incision, First Peoples, Australia, megafauna

## Abstract

Claims have been made about the presumed role of Australia’s First Peoples in the extinction of some of Australia’s megafauna. However, evidence used to suggest butchering may instead demonstrate fossil collection by Australia’s First Peoples. Using micro-computed tomography scanning and microscopic wear analysis, we analysed a cut sthenurine tibia from Mammoth Cave, Western Australia, previously interpreted as evidence of butchering. Our analyses suggest the cut occurred long after death and probably after fossilization. We investigated the possibility of long-distance transportation of a premolar of *Zygomaturus trilobus* gifted by First Peoples in the Kimberley region of Western Australia. This species is otherwise unknown from northern Australia but common in southern Australia. Using X-ray fluorescence, we tested the potential provenance of the premolar and found that it was elementally indistinguishable from Mammoth Cave premolars. These results suggest that First Peoples may have collected fossils in southern Australia before carrying them to the Kimberley region. A review of other recent claims of killing and/or butchering of extinct megafaunal species suggests they too may have been collected as fossils. We argue that fossils were valued, being collected and transported long distances by the First Peoples in Australia in all probability thousands of years before Europeans arrived on this continent.

## 1. Introduction

Interactions between people and extinct megafauna have been demonstrated in many parts of the world, but firm evidence for this in Australia remains controversial. It has been previously suggested that First Peoples in Australia depicted images of extinct species in rock art, drawing on the probability that at least some of Australia’s extinct megafaunal animals overlapped in time and space with Australia’s First Peoples [1–3]. Further, some legends of First Peoples have been interpreted to include stories about interactions between people and some of these extinct megafaunal species [4,5]. There have also been claims that there is hard evidence, in teeth or bones, that some of these species may have been hunted, scavenged or butchered by humans [6] and that these activities may well have contributed to the extinction of these species [7]. Evidence that fossils of extinct species were of interest to and transported by First Peoples has also been demonstrated [8]. It is, however, the sole purpose of our research reported here to reassess claims for hard evidence that any of the Australian extinct megafaunal species, while alive, were in fact killed, scavenged and/or butchered by First Peoples.

**Figure 1.**
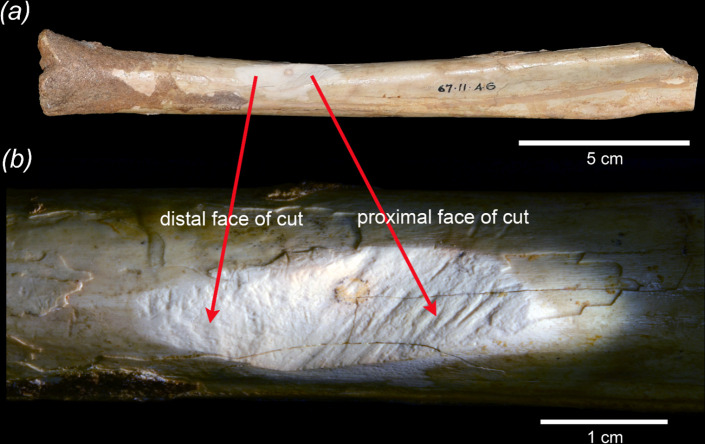
Mammoth Cave tibia (WAM 67.11.46) of an extinct sthenurine kangaroo. Whole fossil specimen (*a*) showing remnants of the CaCO_3_ encrustation (brown) covering much of the distal end of the shaft. The cut (*b*) has two components: the relatively coarsely marked distal face on the left and the relatively finer marked proximal face on the right (see text). Photos: Anna Gillespie.

**Figure 2.**
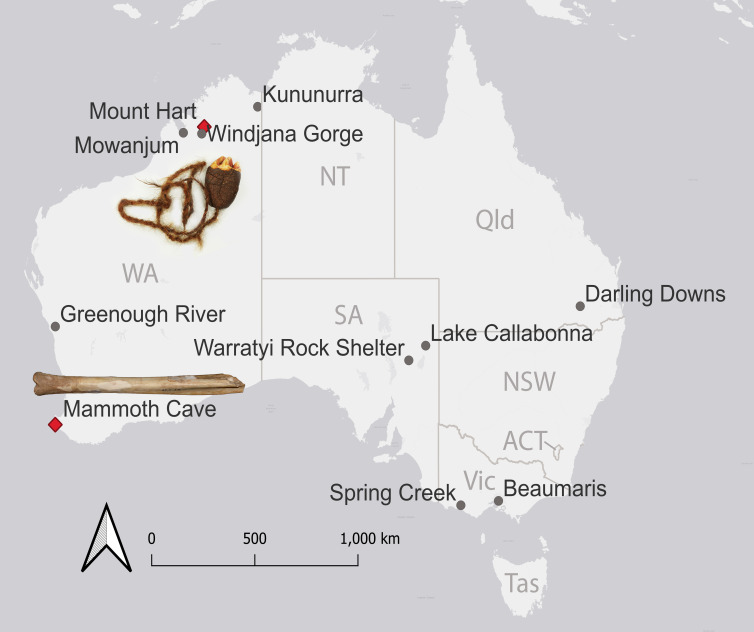
Location of localities noted in this paper. The cut tibia and the *Zygomaturus trilobus* Charm are shown near the areas where they were obtained (Mammoth Cave and Mount Hart, respectively; red symbols). Photos: Anna Gillespie, Helen Ryan and Western Australian Museum. See also figures 1 and 3.

Vertebrate fossils were collected from two sites in Mammoth Cave in southwestern Western Australia between 1909 and 1915 by Ludwig Glauert and Ernest A. Le Souef [6,9,10]. In 1967, while examining postcranial elements of sthenurine kangaroos in these Mammoth Cave collections held by the Western Australian Museum, Archer *et al*. [6] discovered a distinctive, structurally bimodal cut on a partial tibia (WAM 67.11.46) of an extinct sthenurine kangaroo (figures 1 and 2). This partial tibia was identified as being referable to either *Procoptodon browneorum* or *Simosthenurus occidentalis*, diagnostic features that would distinguish tibiae of these two sthenurine species having not yet been established [11].

**Figure 3.**
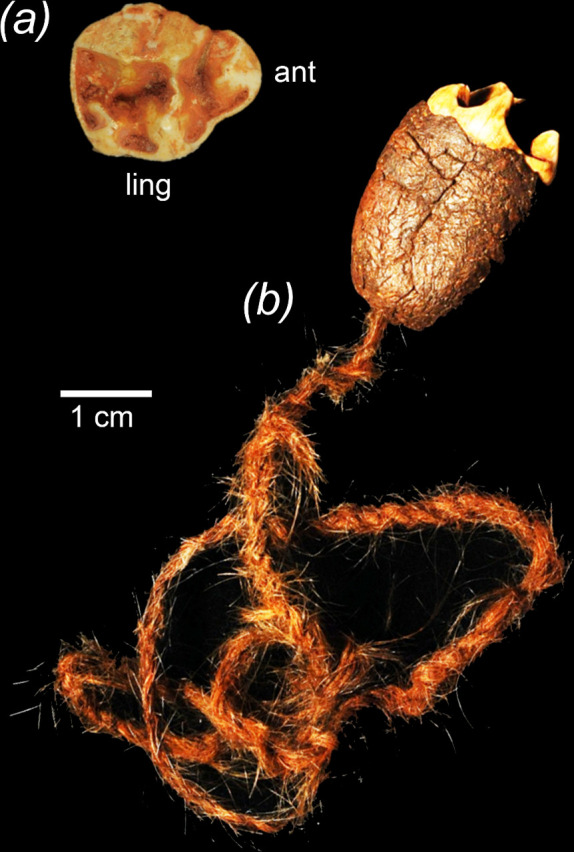
The Kimberley Charm, containing a right upper third premolar (RP^3^) of the extinct diprotodontid *Zygomaturus trilobus* (*a*, occlusal view), mounted in spinifex resin and with attached string made of hair (*b*). Abbreviations: ant, anterior; ling, lingual. Photos: Michael Archer and Western Australian Museum.

An intact continuous CaCO_3_ encrustation was in place over all surfaces of the tibia including the area of the cut. This encrustation, which was removed in 1967, indicated that the cut was made prior to the collection of the specimen by either Glauert or Le Souef. Because the cut is significantly complex and, as concluded by Archer *et al.* [6], not attributable to either animal gnawing or rockfall, it was suggested that it could have been the result of human manipulation that had occurred prior to deposition of the CaCO_3_ encrustation.

Archer *et al.* [6] investigated other potential evidence for human involvement in the accumulation of the Mammoth Cave fossil deposit and tentatively concluded that the cut bone as well as post-mortem changes to other bones in the deposit may well have been the result of human interaction with the bones prior to their interment in the Mammoth Cave fossil deposit. While some of the other marks on Mammoth Cave bones have been suggested by Horton & Wright [12] to possibly be caused by marsupial lions (*Thylacoleo carnifex*), the complex cut that is the focus of this study has none of the features that they would attribute to the actions of this carnivore.

The first attempt to radiometrically date the Glauert Excavation deposit [13] produced a ^14^C date using charcoal fragments of greater than 37 000 years ago (37 ka). A second date, greater than 31.5 ka, also based on ^14^C dating of charcoal, was reported by Merrilees [14]. Roberts *et al.* [15] carried out U/Th dating of the deposit obtaining dates of 44.4 ± 0.64 and 55.2 ± 1 ka. Given current understanding [16] that First Peoples had arrived in Australia around 65 ka, and evidence of human occupation from Devil’s Lair near Mammoth Cave of approximately 50 ka [17], potential overlap in time of living Mammoth Cave sthenurines and humans is therefore probable. In 2010, we revisited the Glauert Excavation in Mammoth Cave and collected CaCO_3_ samples (speleothems) from remnants of the original deposit for further U/Th radiometric dating. The results of this most recent effort to date the deposit are presented below.

Akerman [18] reported an isolated right upper third premolar (P^3^) of the extinct diprotodontid marsupial *Zygomaturus trilobus* mounted in spinifex resin with an attached string made of hair, and four isolated upper cheekteeth of the sthenurine macropodid marsupial *Procoptodon browneorum*. He noted that all of these specimens were said by the First Peoples individuals who gifted them to him in the Kimberley region of northwestern Western Australia to be ‘Charms’ capable of increasing the availability of food. The tawny yellow colour of the tooth enamel and preservation of these teeth resemble others known to have been collected from Mammoth and other caves in southwestern Western Australia.

Akerman [18] noted that the *Z. trilobus* Charm was gifted to him in Derby, Western Australia, by a First Peoples man from Mount Hart in the Kimberley region. He noted that the *P. browneorum* teeth were gifted to him at Mowanjum Mission by a Worora First Peoples man who had most recently been working at Mount Hart but was part of a larger group of First Peoples connected to Mowanjum. These specimens are part of a collection of objects leased to the Western Australian Museum by Buru Energy. Neither of these species is otherwise known from any northern Australia fossil localities, but both are common in cave deposits in southern Australia. Although Akerman [18] noted the possibility that these teeth might have been traded or manuported in other ways up the coast to the Kimberley region from a cave deposit in southwestern Western Australia, he suggested that they were perhaps more likely to have come from an as-yet unknown fossil deposit in the Kimberley region. The location of places noted in this paper is shown in figure 2.

Given the possibility that the *Z. trilobus* premolar, at least, might have been collected from Mammoth Cave or another cave in southwestern Western Australia by First Peoples, non-destructive X-ray fluorescence (XRF) elemental analysis of this tooth was carried out. The same was done with diprotodontid teeth known to be from Mammoth Cave and with teeth known to be from the Kimberley region and elsewhere in Western Australia.

Despite arguments that humans may have directly contributed to the extinction of Australian megafaunal animals during the Pleistocene [19,20], Langley [21] concluded that only the claim by Archer *et al.* [6] based on fossils from Mammoth Cave provided putative hard evidence for human predation and/or scavenging on now-extinct Pleistocene megafaunal species. Hence, that claim merits further testing, which is why we used micro-computed tomography (µCT) and microscopic texture analysis to re-examine the external and internal morphologies of the cut tibia from Mammoth Cave. Other more recent claims for potential hard evidence of human predation or butchering of Australian megafauna are also reviewed here.

## 2. Methods

The Glauert Excavation in Mammoth Cave, southwestern Western Australia, was revisited in 2010 and CaCO_3_ samples (speleothems) were collected from remnants of the original deposit for further radiometric dating. New U-Th radiometric dates were obtained for materials in Mammoth Cave using methods outlined in Hellstrom [22] and published in Woodhead *et al.* [23]. We attempted to directly date the cut sthenurine tibia using accelerated mass spectrometry (AMS) ^14^C dating at ANSTO, Sydney, and to determine a minimum age for the cut itself, we attempted to date the CaCO_3_ crust using U-Th methods at Melbourne University, Melbourne.

To better understand the external and internal structures of the cut bone, it was scanned at a resolution of 33.84 µm on an Inveon Multi-Modality µCT (model 5001). All processing, segmentation, measurement and visualization were performed using Mimics (v. 21, Materialise^®^).

An analysis of microscopic features of both faces of the complex cut was carried out, with scan images produced using a 100× objective on a Sensofar PLu neox optical profiler at Vanderbilt University, Nashville, similar to dental microwear texture analysis [24–26].

Because destructive sampling of the *Z. trilobus* premolar in the Charm was not permitted, non-destructive XRF was carried out (K. Kasi and V. Richards, Materials Conservation Department, Western Australian Museum) using a Bruker AXS Handheld Tracer III-SD SN T3S2520 with channel resolution of 2048 and operating parameters: rhodium tube X-ray source and 10 mm^2^ XFlash^®^ SDD peltier cooled detector with typical resolution of 145 eV at 100 000 counts per second (cps) over an area of approximately 8 mm². All analyses were conducted at 40 kV and 30 μA by using a Cu/Ti/Al (green) filter and an air atmosphere with 600 s live-time count. These settings allow all the X-rays from 17 to 40 keV to reach the sample, thus efficiently exciting the elements from Fe to Mo. There is reduced sensitivity to elements below Fe with these settings. Elements analysed were Ca, Cl, Cr, Cu, Fe, Ga, K, Mn, Ni, P, Pb, Rb, Se, Sn, Sr, Ti, V, Y, Zn and Zr; Ar, Br and Pd were excluded because they were unlikely to occur naturally in the sample.

XRF was performed on the Charm’s *Z. trilobus* premolar as well as 18 *Z. trilobus* premolars from Mammoth Cave (WAM 61.7.17, 61.7.21, 63.7.10−12, 64.10.14−15, 64.10.18, 64.10.22, 64.10.94−95, 64.10.99, 71.12.75−80), one *Z. trilobus* tooth from Greenough River (WAM 77.9.10), a diprotodontid tibia from Windjana Gorge (WAM 71.2.44 a) and a *Nototherium inerme* tooth (another diprotodontid) from Kununurra (WAM 89.6.1), all from Western Australia (figure 2). For each specimen analysed, XRF analysis was reproduced at two different spots on the specimens as a minimum, but up to eight spots were analysed on larger specimens.

A one-way permutational multivariate analysis of variance (PERMANOVA) was performed to test whether the group means were significantly different from one another. A linear discriminant analysis (LDA) was then performed on the multivariate XRF data to group known samples by providence and to predict the providence of the unknown samples. Cross-validation (CV) was used to determine prediction accuracy, though low sample sizes of some locations limited CV accuracy. Prediction was performed on four XRF samples of the Charm using both typicality probabilities and maximum likelihood. Maximum likelihood predictions generate forced allocation, even if samples lie outside notional 95% confidence intervals. Typicality probabilities, however, will reject group prediction if samples lie outside group confidence intervals [27]. LDA was performed in R using the MASS package [28] and typicality prediction using the Morpho package [29].

## 3. Results

### 3.1. New radiometric dating of the Mammoth Cave fossil deposit

**Figure 4.**
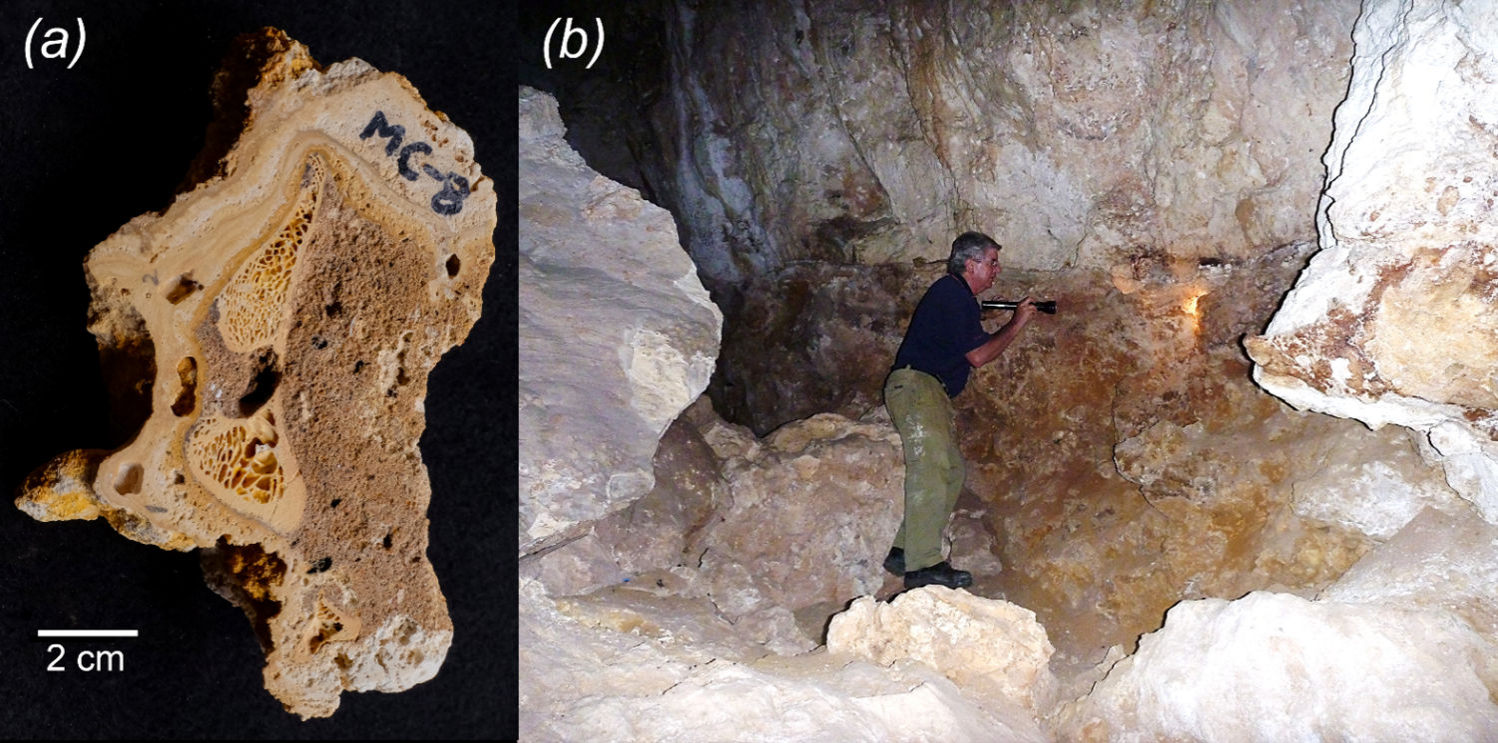
Speleothems (flowstone) coating fossil bone (*a*) from the Glauert Excavation site in Mammoth Cave. These speleothems provided a radiometric date of 49 ± 2 ka. Photo: Jon Woodhead. Glauert Excavation site (*b*) with the late Lindsay Hatcher’s torchlight indicating from where the sample was obtained. The aeolianite cave wall above and behind the brownish fossil deposit was also dated as was a broken stalactite adjacent to this site. Photo: Michael Archer.

New U-Th radiometric dates were obtained for materials in Mammoth Cave (figure 4) using methods outlined in Hellstrom [22] and published in Woodhead *et al.* [23]. Samples of speleothems coating the cave wall behind and hence in part confining the fossil deposit excavated by Glauert provided a date of 122 ± 16 ka. A date for broken pieces of young stalagmites found near but not in the deposit was 0.97 ± 0.06 ka. Dates for speleothem growth laminating a fossil bone (figure 2) exposed in the deposit were 52 ± 1 and 53 ± 2 ka. Considering these dates and previous U-Th radiometric dates obtained for the same deposit by Roberts *et al.* [15] of 44.4 ± 0.64 and 55.2 ± 1 ka, it is probable that at least some of this fossil deposit was accumulating after the time of arrival of people into Australia around 65 ka [16]. Attempts to directly date the cut sthenurine tibia were unsuccessful, because insufficient collagen was present in the bone itself, using the assessment methodology of Brock *et al.* [30]. Attempts to date the CaCO_3_ crust as a way of determining a minimum age for the cut were also unsuccessful because this material was too porous (an ‘open system’) such that no age could be determined.

### 3.2. Reassessment of the cut on the diaphysis of tibia WAM 67.11.46

**Figure 5.**
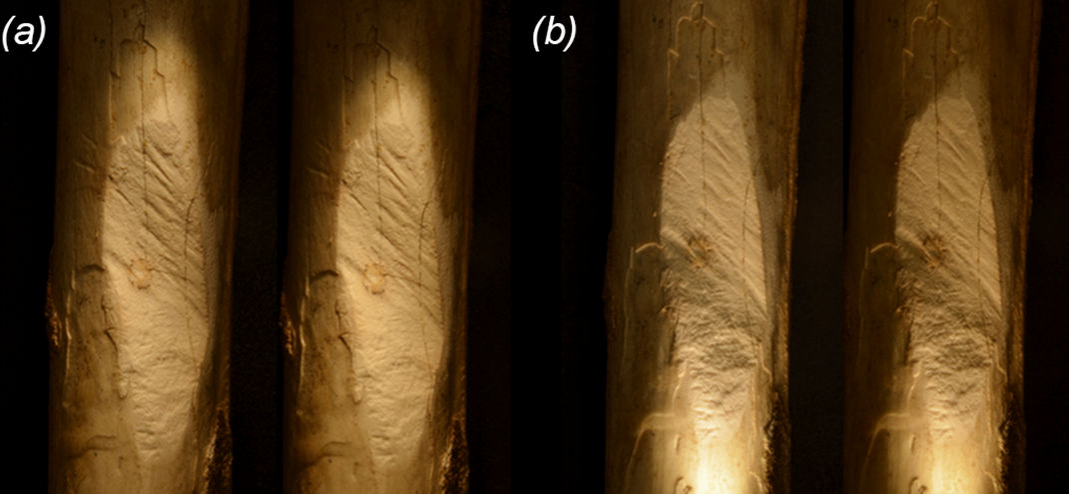
Stereopair macroscopic images of the two faces of the cut in WAM 67.11.46. In low-angle lighting, focus is on the proximal face (top half) of the cut (*a*) and the distal face (bottom half) of the cut (*b*). Photos: Anna Gillespie.

Archer *et al.* [6] described the cut found on the incomplete, subadult sthenurine tibia (the epiphysis was not fixed to the diaphysis) that had been revealed when the CaCO_3_ encrustation that covered the cut area and most of the rest of the bone was removed during initial investigation in 1970. As noted in that description [6], the cut has two distinctly different adjacent faces incised into the diaphysis closer to the distal than the broken proximal end of the tibia (figure 5). These faces meet at an angle of approximately 130°. The maximum depth of the incision from what had been the surface of the shaft is 3 mm. The incision markings on both faces are oblique to the long axis of the shaft. The marks on both faces appear to be inclined approximately 30° to the long axis of the shaft, but in opposite directions, those on the distal face being distally inclined while those on the proximal face are proximally inclined. The orientation of the incision marks on the two faces also differs, with those on the distal face being more or less linear and parallel, while the marks on the proximal face are less regular and in general appear ‘battered’. For these reasons, it was concluded by Archer *et al.* [6] that a single action, such as a stone falling on the shaft, could not have caused the cut. The fact that both faces are more or less planar and with different types of incision details (see below) also argues against a rockfall or any single impact as the cause. The fact that there is no comparable mark or depression on the opposite side of the shaft suggests the possibility that the cut was inflicted on the bone while it was still held by and projecting from the fossil deposit in an effort to obtain that projecting part of the bone.

Following experiments with and research on the nature of marks on bones caused by Australian ossivorous or carnivorous mammals, Archer *et al.* [6] concluded that the cut was not caused by an animal gnawing or chewing on the tibia. Among other reasons for this conclusion was that there are no ‘bracing marks’ on the other side of the shaft behind the cut that made dental incursion by a relatively large animal such as *Sarcophilus harrisii* or *T. carnifex* improbable (for examples of tooth-marked bones from these species, see Sobbe [31] and Horton & Wright [12]). Rodent gnawings are also an improbable cause because of the planar rather than scooped surfaces of both faces. Further, rodents primarily use their lower incisors when gnawing, which results in shallow U-shaped incisions more or less at right angles to the long axis of the area of bone being gnawed, decidedly unlike the cut faces on the sthenurine tibia. Further, rodents tend to leave upper incisor ‘anchor’ marks at a distance from the gnawed area.

**Figure 6.**
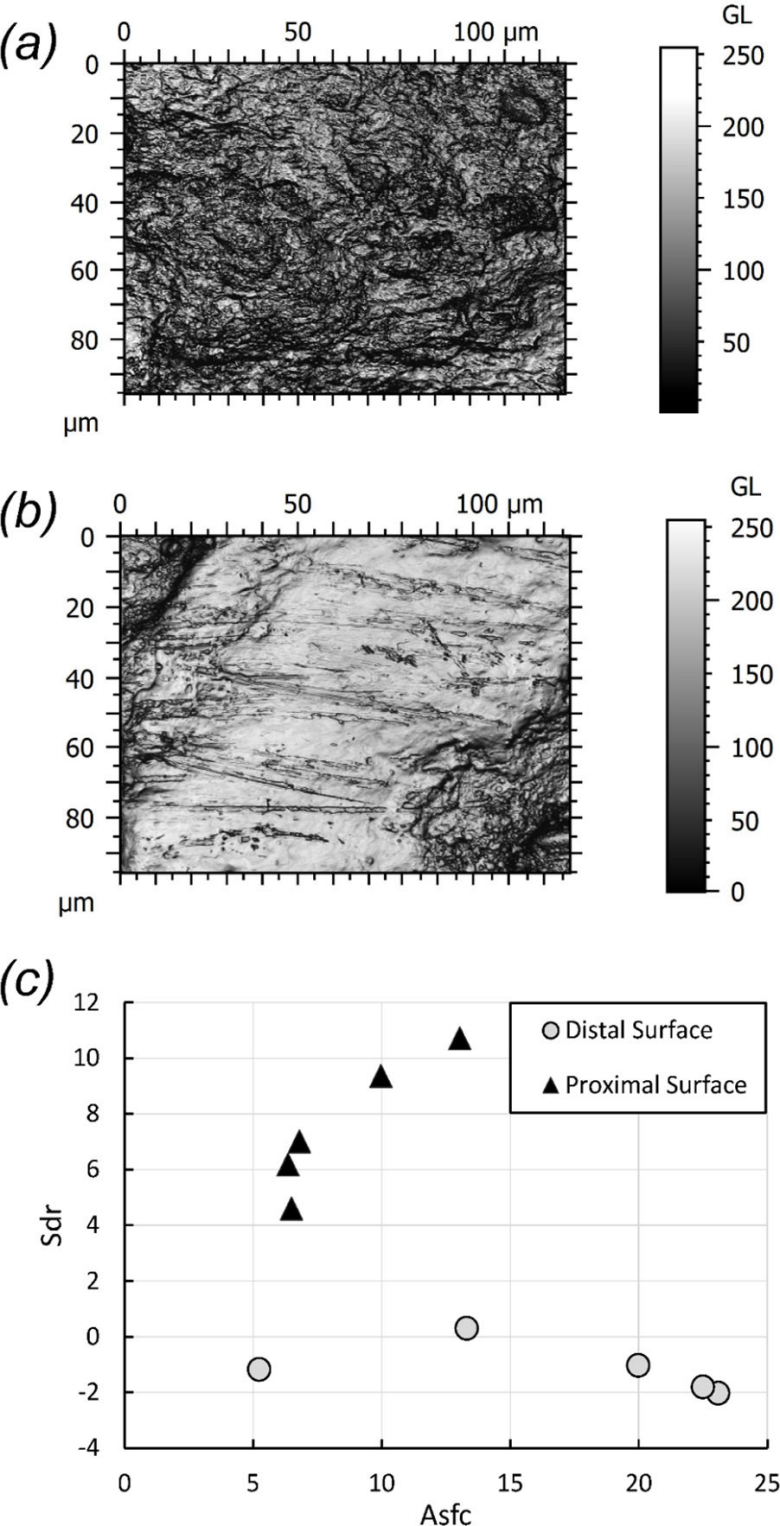
Examples of microscopic wear features on the distal face (*a*) and proximal face (*b*) from specimen WAM 67.11.46. Textural attributes (Sdr and Asfc) characterizing the surfaces of the two faces are shown in (*c*). The relatively chaotic, nonlinear cuts in the distal surface (*a*) clearly contrast with the relatively more uniform, less complex linear cuts in the proximal surface (b).

Analysis of microscopic features involved a range of different conditions in the surfaces of the two cut faces (figure 6). The distal face is more complex (higher Asfc) with larger/deeper pits and more homogeneous in complexity than the proximal face (lower HAsfc_2×2_ to HAsfc_11×11_); this pattern is observed at all scales and is significant at most (electronic supplementary material, table S1). Further, anisotropy (epLsar) is higher on the proximal face as compared to the distal face—thus, the directionality of the features is observable at the microscopic level (figure 6), in addition to macroscopically (figure 5). In terms of ISO height parameters, kurtosis of the height distribution (Sku) is lower on the distal face than on the proximal face, although both faces have spiked distributions. Skewness of height (Ssk) has lower values on the proximal face than on the distal face. Both of the hybrid textural parameters, the root mean square gradient (Sdq) and developed interfacial area ratio (Sdr), are lower on the proximal face, though only Sdr approaches significance (electronic supplementary material, table S1). In terms of volume, Dale void volume at a given height (Vvv) is higher on the proximal face than on the distal face. These microscopic textural differences have been interpreted by us to mean that it is probable that the two cut faces were produced by different mechanisms rather than the result of one process acting simultaneously to create both faces.

### 3.3. Micro-computed tomography analysis of the diaphysis of WAM 67.11.46

**Figure 7.**
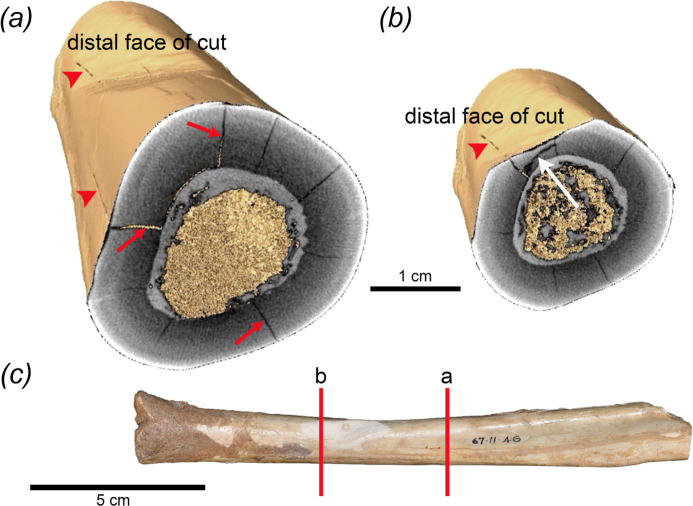
Cross sections revealing internal fractures detected by µCT imaging of WAM 67.11.46. Three of nine longitudinal cracks are indicated by red arrows in the cross section (*a*). The external expression of one of the longitudinal cracks is indicated by red arrowheads along the surface of the bone. Within the area of the cut (*b*), the single transverse fracture (white arrow) is terminated at each end by two longitudinal cracks indicating that its development post-dated that of the longitudinal cracks. Approximate positions of cross sections (*a*) and (*b*) in WAM 67.11.46 (*c*). Photo in (*c*) by Anna Gillespie.

µCT scanning reveals that the shaft has at least nine deep longitudinal cracks (figure 7*a*; electronic supplementary material, video) that extend along the diaphysis. Two of these pass through the area that has been incised. Each transects the bone from the edge of the medullary cavity towards the external surface of the bone. At least some of these reach the external surface and are visible on the outer surface of the diaphysis. All of these appear to be shrinkage cracks that developed long after the animal was dead. Importantly, there is also one short transverse crack within the area of the cut that extends between two of the longitudinal cracks (figure 7*b*). Because it is truncated at both ends where it meets these two longitudinal cracks, it must have developed at a time later than that when the longitudinal tracks developed. Further, because it occurs within the laminar bone matrix of the shaft within the area of the cut, it seems probable that this transverse crack resulted from the same forces that were involved in producing the incision itself. Consequently, the date of the incision must have post-dated dehydration of the shaft, i.e. the incision did not occur when the bone was still fresh, in contrast to the conclusion reached by Archer *et al.* [6] Although Archer *et al.* [6] argued that the small, discoloured spot near the base of the incision may have been caused by staining from organic fluids within the lumen of the shaft, the µCT image demonstrates that the base of the incision had only reached about halfway through the laminar bone. Therefore, it now seems that even if the incision had occurred when the bone was fresh, staining of the base of the cut through the undisturbed layers of laminar bone would have been improbable if not impossible.

With regard to the fact that the cut surfaces of the tibia appear to be basically the same colour as the undisturbed surfaces of the shaft itself, we consider that this reflects the fact that the overall colour of the fossil bones from Mammoth Cave is a uniform tawny yellow throughout the thickness of the cortical bone. Hence, the similarity in colour of the cut surface and the shaft itself is not a basis for doubting that the cut occurred some time after the bone had become fossilized or at least sufficiently dehydrated to develop the series of longitudinal cracks that preceded the incision.

Although the relationship of the transverse crack associated with the cut and the longitudinal drying cracks indicates that the former occurred after the latter developed, the amount of time that elapsed between the two processes is not clear given uncertainty about how long it may have taken to develop the longitudinal cracks through natural degradation. Hence, although the incision occurred on this bone long after death and after dehydration of the bone, it could have occurred prior to the bone becoming an integral part of the fossil deposit from which it was obtained by palaeontologists.

### 3.4. Geochemical analysis of the *Zygomaturus trilobus* premolar in the Kimberley Charm

The XRF analysis of the right upper premolar (RP^3^) of *Z. trilobus* that is part of the Kimberley Charm (figure 3) provided results as follows.

**Figure 8.**
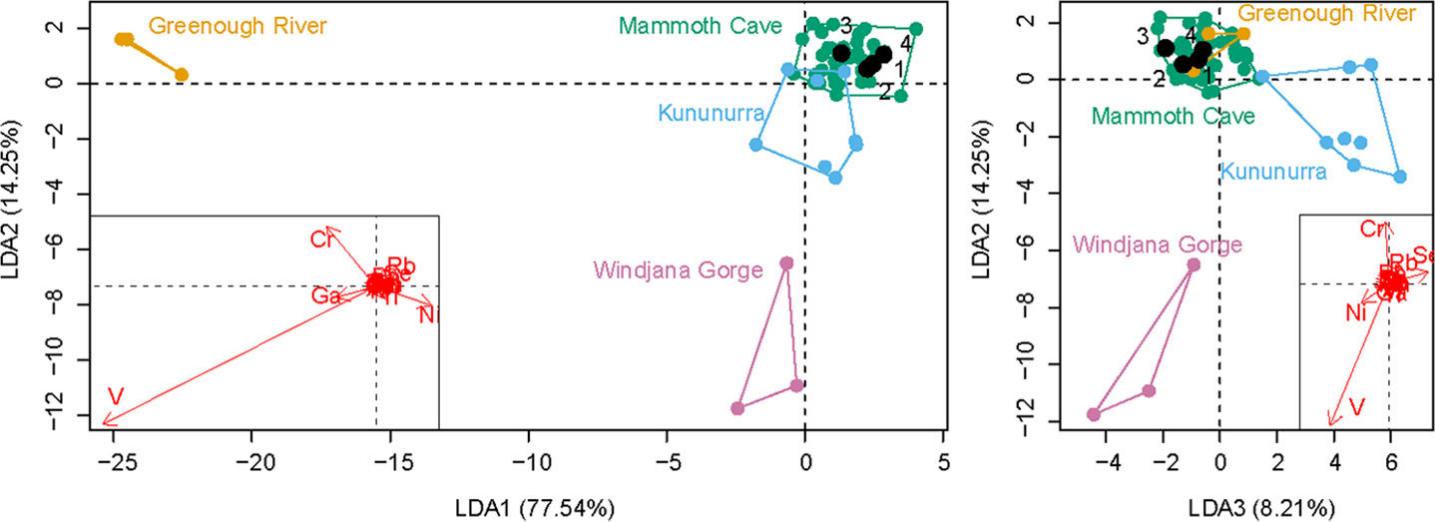
Plot of the results of the LDA analysis, with group convex hulls drawn. Black numbered points represent samples of the Charm and correspond to samples listed in table 3. Mammoth Cave = 18 individual P^3^ teeth of *Z. trilobus* from Mammoth Cave. Greenough River = a P^3^ of *Z. trilobus* from Greenough River. Kununurra = a tooth of *Nototherium inerme* from a fossil deposit in the Kimberley region. Windjana Gorge = a diprotodontid tibia from Windjana Gorge.

Ca was the most abundant element among all samples, followed by Sr and Y, though these did not provide the best discrimination among sites (see electronic supplementary material, table S2, for raw and normalized XRF elemental compositions, and electronic supplementary material, figure S1, for site means). Instead, trace elements V, Ga, Cr, Ni and Se provided the best discrimination using LDA (figure 8). LDA (or the generalized multivariate form) is the standard multivariate method for discrimination and allocation across fields. LDA classification accuracy was 94.83%, and CV found all groups reliably classified correctly. Only Windjana Gorge returned a notable error—incorrectly classifying as Mammoth Cave 33% of the time, though the inverse was not true: Mammoth Cave was correctly classified 98% of the time (see table 1 for the full confusion matrix).

**Table 1.** Confusion matrix and classification probabilities of the linear discriminant analysis between *a priori* groups. Classification accuracy of each group is in bold along the diagonal.

	Greenough River	Kununurra	Mammoth Cave	Windjana Gorge
Greenough River	**1**	0	0	0
Kununurra	0	**0.875**	0.125	0
Mammoth Cave	0	0.022727	**0.977273**	0
Windjana Gorge	0	0	0.333333	**0.666667**

LD Axis 1 accounted for 77.54% of the variance, loading strongest with V, Ni, Cr and Ga. Axis 2 accounted for 14.25% of the variation, loaded primarily by V and Cr. Finally, Axis 3 accounted for 8.21% of the variation, loading strongly with V and Se. Greenough River was discriminated along LD1, based on particularly high levels of V, followed by Cr and Ga, separating it strongly from the other three sites along LD1. Windjana Gorge was discriminated along LD2 based also on high levels of V, and moderate amounts of Ni and Ga. Mammoth Cave and Kununurra were the most similar and were separated along LD3. Kununurra was discriminated based on high levels of Se, while Mammoth Cave was particularly high in V and Ni.

Classification of the Charm in this LDA (table 2) found strong support for a Mammoth Cave affinity, with universal support by posterior probabilities, and three of the four samples supported by typicality probabilities. PERMANOVA found similar results (table 3) with significant overlap between the Charm and the Mammoth Cave (*p* > 0.05).

**Table 2.** Classification of Charm provenance by typicality and posterior probabilities. Values in bold are classifications statistically indistinguishable from the site of provenance (*p* > 0.05). Abbreviations: typ. = provenance by typicality probability; lik. = provenance by posterior probability.

	Greenough River *Z. t*.		Kununurra		Mammoth Cave *Z. t*.		Windjana Gorge	
Charm P^3^	typ.	lik.	typ.	lik.	typ.	lik.	typ.	lik.
sample 1	0.000	0.000	0.001	0.000	**0.979**	**1**	0.000	0.000
sample 2	0.000	0.000	0.000	0.000	**0.914**	**1**	0.000	0.000
sample 3	0.000	0.000	0.000	0.000	0.036	**1**	0.000	0.000
sample 4	0.000	0.000	0.001	0.000	**0.973**	**1**	0.000	0.000

**Table 3.** Pairwise comparison of the one-way PERMANOVA. Pairwise comparisons in bold are statistically indistinguishable from one another (*p* > 0.05).

	Greenough River *Z. t*.	Mammoth Cave *Z. t*.	Kununurra	Windjana Gorge	Charm P^3^
Greenough River *Z. t*.		0.0001	0.0065	**0.0965**	0.0258
Mammoth Cave *Z. t*.	0.0001		0.0001	0.0379	**0.8322**
Kununurra	0.0065	0.0001		0.0057	0.0019
Windjana Gorge	**0.0965**	0.0379	0.0057		0.0303
Charm P^3^	0.0258	**0.8322**	0.0019	0.0303	

The primary elemental data obtained via the XRF analyses and a histogram plot of the values are given in electronic supplementary materials, table S2 and figure S1).

Despite the strong evidence above, we are cautious about making definitive conclusions about the provenance of the *Zygomaturus* P^3^ in the Kimberley Charm. Surface XRF would have introduced error through sample heterogeneity and surface topology, probably significantly limiting our discriminatory power. Further, limited sampling of localities reduces the granularity of possible sites of provenance. However, despite the presence of within-sample variance, LDA classification accuracy was high at 94.83%. Further, while drawing conclusions directly from maximum likelihood would be biased to only the sites sampled, typicality probabilities retain the uncertainty of rejecting membership to these groups. As such, the classification of three of the Kimberley Charm samples as Mammoth Cave by typicality suggests a very strong similarity in composition. Ultimately, the results of the XRF analysis reveal a tight overlap between the *Z. trilobus* premolar in the Kimberley Charm and the *Z. trilobus* premolars from Mammoth Cave and do not reject the possibility that the Kimberley tooth may have come from the Mammoth Cave deposit. However, this cannot be used as evidence that that is where it necessarily came from.

It could be important in the future, if and when circumstances allow, to investigate the isotopic composition (e.g. strontium, oxygen) of the *Z. trilobus* tooth within the Charm as a further means to help determine the geographic/geologic origins of this tooth within the Australian landscape [32–35]. Although such information might provide a useful test of the hypothesis that we present in the current study, for cultural reasons, we have not been permitted to undertake destructive analysis of the tooth itself, thus limiting our work to non-destructive XRF analysis, the results of which are presented in this paper.

## 4. Discussion

We have demonstrated that the cut on the Mammoth Cave sthenurine tibia was in all probability caused by tool-using humans acting on the bone after it had become dehydrated or more likely fossilized, in contrast to the conclusions of Archer *et al.* [6]. We suggest here that the purpose of this effort may have been the retrieval of fossils from the bone-rich, late Pleistocene deposit in Mammoth Cave after its discovery by First Peoples. When this occurred is not clear, but it may have been at some time after the fossil deposit was formed, which could have been as long ago as approximately 55 ka [23].

However, there are claims that First Peoples in Sahul were responsible for the extinction of megafaunal species either by over-hunting [36], use of fire [37] or perhaps a combination of these factors [19,20] soon after their arrival. While these are hypotheses, hard evidence is required before it can be concluded that predation on the now extinct megafaunal species by First Peoples occurred. Despite this, some researchers have paradoxically implied that the lack of evidence for anthropogenic predation may support a primary role for humans in over-hunting some species to extinction [7].

Many arguments have been put forward [38–42] to alternatively conclude that natural changes in the Australian environment associated with climate change were as likely if not more so to be the cause of at least some of the megafaunal extinctions. This may be supported by the fact that numerous species [43–46] have long temporal overlaps with the continent’s earliest peoples (thus ruling out rapid extinction following human arrival [16]), with several dropping out of the fossil record at times of climate stress [38]. Yet others have argued [47] that climate change constrained regional habitats, exacerbating whatever impact humans may have had on relatively confined areas of habitat.

Following detailed investigations by others, justifiable claims for hard evidence that First Peoples hunted or scavenged even a single individual of any of the now extinct megafaunal species are in significant doubt. Four claims of this kind are current: Archer *et al.* [6] focused on the cut sthenurine tibia from Mammoth Cave, Western Australia; Vanderwal & Fullagar [48] focused on a marked incisor of *Diprotodon optatum* from Spring Creek, Victoria; Miller *et al.* [49] focused on bird eggshell interpreted by them to represent the flightless, megafaunal dromornithid bird *Genyornis newtoni*; and Hamm *et al.* [50] focused on a radius fragment of *D. optatum* from Warratyi Rock Shelter, South Australia.

In the case of the Mammoth Cave bone [6], we have presented evidence herein demonstrating that the damage to the sthenurine tibia did not occur until after the bone had become severely dehydrated or more probably fossilized.

Considering the Spring Creek *D. optatum* incisor [48], Langley [21] has concluded that the cuts on that tooth were in fact made by tiger quolls (*Dasyurus maculatus*), not people.

In regard to the megafaunal bird eggshell claim [51,52], Grellet-Tinner *et al.* [53] argued that the eggshell fragments examined by Miller *et al.* [51,52] are much too small to have been produced by the dromornithid megafaunal bird *Genyornis newtoni* and were probably the eggs of a far smaller, non-megafaunal megapode bird. The claim was challenged [53] partly on the basis that the eggshell fragments differed in some details from some megapode species leaving the possibility that other megapodes among the many known may have (or have had) eggshells similar to those being attributed to *G. newtoni* [51,52]. Independently, it was concluded [54] that the putative *G. newtoni* eggshell remains examined by Miller *et al.* [51,52] were more likely to have been produced by megapode birds than *G. newtoni*. However, it has also been argued [55] that protein analysis of some of these eggshell fragments suggests some may have been produced by a galloanseran bird, a very diverse order that includes dromornithids as well as other groups of birds such as swans, geese, ducks and brushturkeys. Ongoing arguments about the biological origin of these eggshell fragments suggest they are not conclusive evidence that First Peoples were responsible for exterminating this or any other megafaunal Australian bird.

A heavily degraded *Diprotodon optatum* radius fragment (EMU RS-6737-7754) that was obtained from a level in the Warratyi Rock shelter dated at greater than 46 ka [50] has been argued to be unambiguous evidence of the hunting of megafauna by humans [50]. However, this conflicts with remarks in Hamm *et al.* [50] (electronic supplementary material, p. 46) that state: ‘The marks on the Warratyi bone [which are consistent with the activities of invertebrates and microbes] are broad and flat … and cannot be confused with stone tool cut marks. Other modifications such as carnivore and impact marks due to hammer blows can also be expected in archaeological sites. None of these features appear on the Warratyi bone that would support the case for human modification’.

It was also argued [50] that the high elevation of the shelter above the valley floor made it improbable that a *D. optatum* could have climbed up to the shelter, and the absence of chewing marks indicated that it was not carried in by a carnivore. We note, however, that 46 ka the valley floor would probably have been considerably higher than it is now, and the shelter area may in fact have been accessible to an individual of this species at this time.

None of the specimen-based observations noted above demonstrate that the Warratyi *D. optatum* humerus fragment had been obtained from a living individual that had been killed or butchered by First Peoples. However, it remains possible that the Warratyi *D. optatum* humeral fragment could have been manuported to the rock shelter in the Flinders Ranges, as an intrinsically interesting fossil. This bone could have been picked up from any number of other localities for this widely dispersed, once common species [56], and it is noteworthy that many well-exposed, 50−54 ka skeletons of *D. optatum* [57,58] are exposed on the surface of Lake Callabonna, a fossil-rich lake-bed within 120 km from Warratyi.

Several other examples of First Peoples having collected and manuported fossils in Australia have previously been documented. Whitehouse [8] documented cases involving transported fossil trilobites and bivalves as well as ammonites. Akerman [18] noted that along with the marsupial teeth he was gifted in the Kimberley were other fossils including molluscan belemnites. Rich *et al.* [59] suggested the possibility that a geographically and stratigraphically anomalous dentary (NMV P16279) of a diprotodontid marsupial referable to *Kolopsis* sp. cf. *K. torus* may have been carried by First Peoples from Beaumaris in Victoria to where it was subsequently found by European collectors on the Darling Downs in Queensland, where there are otherwise no known deposits that could have produced this specimen. Spriggs & Russell [60] review evidence that First Peoples in Queensland and New South Wales knew that bones of megafaunal animals recovered by Europeans from caves did not belong to living animals.

First Peoples on other continents such as Africa are similarly known [61] to have keen interests in and collected fossils. Chinese First Peoples are also known to have collected fossils regarded to be ‘Dragon Bones’ at least 120 BC [62]. Palaeolithic hominins were similarly known to appreciate fossils [63]. Given cultural awareness of the morphology of teeth and skeletal elements in living species, fossils that revealed unfamiliar features would have triggered interest and probably an interpretation that these came from pre-existing real or mythical creatures on those continents. Celebrated European palaeontologists arriving in Australia after 1788, such as Ludvig Glauert, Gerard Krefft and Charles Darwin, had the same interests for similar reasons. However, in view of the evidence discussed in this paper, we would argue that these post-1788 scientists were clearly not the first people to have a strong interest in thinking about and collecting fossils in Australia.

## 5. Conclusions

We conclude that the cut on the Mammoth Cave sthenurine tibia, which was originally regarded [6] to be evidence that First Peoples had either killed or butchered this individual, was indeed produced by human actions but on a probably already fossilized bone. This adds to previous indications that First Peoples were interested in and collected fossils. Further, analysis of the *Z. trilobus* tooth, which is part of a Charm obtained from the Kimberley region of Western Australia, suggests the possibility that southern Australian fossils were being manuported via trade routes to northern Australia. Finally, our review challenges claims that there is any hard evidence that First Peoples killed or butchered any individual of any of the now extinct megafaunal species in Australia. This is not to say that it did not happen, just that there is now no hard evidence to support that it did. What we can conclude is that the first people in Australia who demonstrated a keen interest in and collected fossils were First Peoples, probably thousands of years before Europeans set foot on this continent.

## Data Availability

There are no additional data other than those presented in the main document and the supplementary materials [64].
